# AcbHLH144 transcription factor negatively regulates phenolic biosynthesis to modulate pineapple internal browning

**DOI:** 10.1093/hr/uhad185

**Published:** 2023-09-07

**Authors:** Qian Li, Guang Wang, Ling Zhang, Shijiang Zhu

**Affiliations:** Guangdong Province Key Laboratory of Postharvest Physiology and Technology of Fruit and Vegetables, College of Horticulture, South China Agricultural University, Guangzhou 510642, China; Guangdong Province Key Laboratory of Postharvest Physiology and Technology of Fruit and Vegetables, College of Horticulture, South China Agricultural University, Guangzhou 510642, China; Guangdong Province Key Laboratory of Postharvest Physiology and Technology of Fruit and Vegetables, College of Horticulture, South China Agricultural University, Guangzhou 510642, China; Guangdong Province Key Laboratory of Postharvest Physiology and Technology of Fruit and Vegetables, College of Horticulture, South China Agricultural University, Guangzhou 510642, China

## Abstract

Internal browning (IB), a major physiological disorder of pineapples, usually happens in postharvest processes, but the underlying mechanism remains elusive. The bHLH transcription factors are involved in regulating various biological processes, but whether they could regulate tissue browning in fruit during storage remains unknown. Here we showed that the phenolic biosynthesis pathway was activated in pineapples showing IB following 9 days of storage. *AcbHLH144* expression was the highest of the 180 transcription factors identified, downregulated in pineapple with IB, and negatively correlated with the major phenolic biosynthetic genes. AcbHLH144 was shown to be localized in the nucleus and its transient overexpression in pineapples and overexpression in *Arabidopsis* decreased phenolic biosynthesis. The yeast one-hybrid assay and electrophoretic mobility shift assay showed that AcbHLH144 directly bound to the *Ac4CL5* promoter and the dual-luciferase reporter assay showed that it inactivated *Ac4CL5* transcription. These results strongly suggest AcbHLH144 as a repressor for phenolic biosynthesis. Abscisic acid (ABA) alleviated IB, reduced phenolic accumulation, and downregulated phenolic biosynthetic genes, including *Ac4CL5*. Transcriptomic analysis showed that *AcbHLH144* was the most upregulated of all 39 bHLHs in response to ABA. ABA enhanced *AcbHLH144* expression, reduced phenolic contents, and downregulated phenolic biosynthetic genes in pineapples transiently overexpressing *AcbHLH144.* Moreover, ABA enhanced enzyme activity of GUS driven by the *AcbHLH144* promoter. These results showed that AcbHLH144 as a repressor for phenolic biosynthesis could be activated by ABA. Collectively, the work demonstrated that AcbHLH144 negatively regulated phenolic biosynthesis via inactivating *Ac4CL5* transcription to modulate pineapple IB. The findings provide novel insight into the role of AcbHLH144 in modulating pineapple IB during postharvest processes.

## Introduction

Tissue browning poses a severe quality problem for different fruits and vegetables, such as pineapple [1], eggplant [2], peach [3], apple [4], longan [5], and litchi [6]. Phenolic compounds and oxidative enzymes may be the most important biochemical factors that affect tissue browning in eggplant [7]. However, the extent of browning is related to phenolic compound content and not to polyphenol oxidase (PPO) activity in apple [4]. In fact, phenolic compounds are positively correlated with browning severity in three carrot cultivars [8], in a collection of eggplant hybrids and varieties [9], and in fresh-cut apples of 23 cultivars [4]. These findings suggest a key role of accumulation of phenolic compounds in tissue browning development.

Most biological processes are finely regulated by transcription factors (TFs) in eukaryotic organisms [10]. The basic helix–loop–helix (bHLH) TF family is one of the largest TF families found in eukaryotic organisms [11]. The bHLHs are involved in regulating a wide array of plant biological processes and responses to environmental stresses, including drought and salt tolerance [12], fruit ripening [13], cotton fiber elongation [14], iron deficiency tolerance [15], and chilling resistance [16]. In particular, the bHLH TFs participate in regulating secondary metabolites produced via the phenylpropanoid pathway. Overexpressing grapevine *VvbHLH1* in *Arabidopsis thaliana* increases the content of flavonoids [17]. The MYB-bHLH-WD repeat complexes regulate flavonoid biosynthesis [18]. MYB-bHLH-TTG1 complexes regulate anthocyanin accumulation in developing *A. thaliana* seedlings [19]. CmHLB promotes the expression of lignin biosynthetic genes to enhance the lignin content in chrysanthemum [20]. GhbHLH18 negatively regulates cotton fiber strength and length by enhancing biosynthesis of lignin [21]. Overexpression of *OsbHLH034* positively activates lignin biosynthesis in rice [22]. SmbHLH60 functions as a repressor in regulating the biosynthesis of anthocyanins [23]. Phenolic compounds, an important category of secondary metabolites of fruit and vegetables [4], can be regarded as the precursors of flavonoids, anthocyanin, and lignin, as they are produced in the initial steps of the phenylpropanoid pathway [24]. In *Salvia miltiorrhiza* hairy roots, overexpression of *SmbHLH148* [25] and *SmJRB1* [26] increased phenolic acids, the major active metabolites in the Chinese herb, while overexpression of *SmbHLH60* [23] and *SmbHLH3* [27] reduced phenolic acids. However, whether and how a bHLH TF is involved in regulating tissue browning-related phenolic biosynthesis in harvested fruit proceeding to senescence remains elusive.

Some plant biological responses mediated by plant hormones involve bHLH TFs. CES (a bHLH) binds to the promoter of *GA2ox7* to enhance its expression in response to brassinosteroid [28]. Jasmonic acid-mediated seed production and stamen development are regulated by a bHLH–MYB complex [29]. EjbHLH14 plays a role in methyl jasmonate (MeJA)-mediated inhibition of lignin accumulation in loquat at low temperature [30]. We showed previously that abscisic acid (ABA) suppressed internal browning (IB) of harvested pineapples [1], but whether that process involves the regulation of a bHLH TF remains unknown.

Pineapple [*Ananas comosus* L. (Merr.)], a herbaceous fruit produced in tropical and subtropical regions, is of great economic importance to the local agricultural industry. However, the biggest problem that the pineapple industry faces is IB, also referred to as blackheart, a physiological disorder usually happening to harvested pineapple fruit that proceeds to senescence. The IB severely reduces fruit quality with no obvious exterior symptoms and often causes heavy losses in producing countries [1]. In this study, a pineapple bHLH, AcbHLH144, was identified and we characterized its role in regulating phenolic compounds in relation to IB occurrence and in response to ABA. The objective of this work was to unravel the mechanism underlying phenolic biosynthesis in relation to IB development regulated by AcbHLH144. As previous studies on the roles of bHLH TFs were mainly conducted using developing plants, the present work may enhance understanding of bHLH TFs’ roles in modulating pineapple IB during postharvest processes.

## Results

### Phenolic compounds accumulated in pineapple fruit with internal browning symptoms

**Figure 1:**
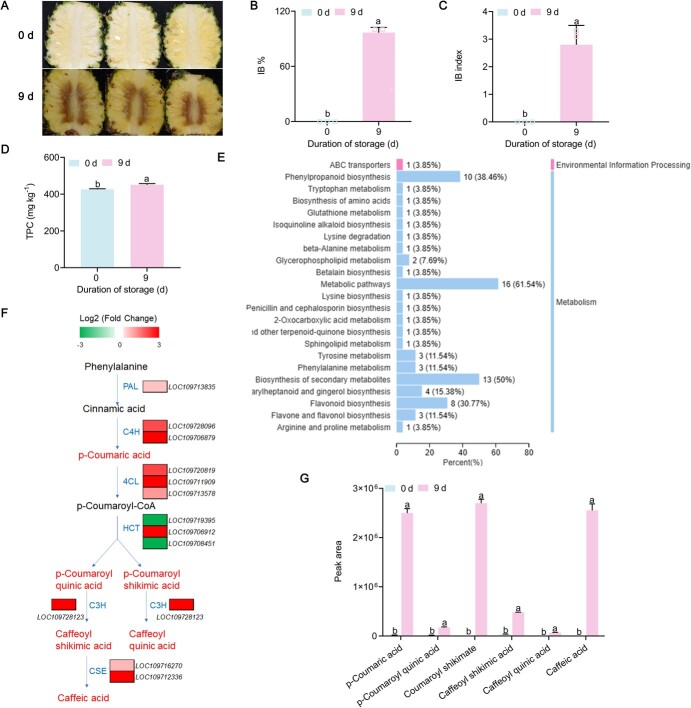
Changes in phenolic compounds in pineapple with IB caused by 9 days of storage. **A** IB symptoms in pineapples after being stored for 9 days. **B** Incidence of IB. **C**: Index of IB. **D** Total phenolic compounds (TPC). **E** Top 23 KEGG enrichment pathways of differentially accumulated metabolites (DAMs) in stored pineapples in comparison with pineapples without storage. **F** Reconstruction of phenylpropanoid biosynthesis pathway highlighting phenolic acid biosynthesis with DEGs and DAMs. Color of charts for DEGs represents upregulation (red) and downregulation (green). Font color for DAMs: green, downregulation; black, no significant change. *AcPAL*, phenylalanine ammonialyase; *AcC4H*, cinnamate 4-hydroxylase; *Ac4CL*, 4-coumarate CoA ligase; *AcHCT*, hydroxycinnamoyl-CoA:shikimate/quinate hydroxycinnamoyl transferase; *AcC3′H*, 5-*O*-(4-coumaroyl)-d-quinate 3′-monooxygenase; *AcCSE*, caffeoyl shikimate esterase. Font color for DAMs: red, upregulation; black, no significant change. **G** Relative abundance (peak area) of each of the six phenolic acids in stored vs non-stored fruit. Significant differences are shown by letters above the bars (*P* < .05).

The severity of pineapple IB is usually measured by two parameters, IB incidence (referring to the percentage of fruit with IB) and IB index (referring to the average rating of IB severity) [1]. In this study, pineapple fruit stored for 9 days exhibited severe symptoms of IB (Fig. 1A), with the IB incidence and index being 100% (Fig. 1B) and 2.8 (Fig. 1C), respectively. The browning of fruit has been shown to be linked to phenolic compound accumulation [4, 8]. Here, pineapple with IB following 9 days of storage had higher total phenolic compounds (TPC) (Fig. 1D). Metabolomics analysis revealed that phenylpropanoid biosynthesis was among the top three of the 23 most enriched KEGG (Kyoto Encyclopedia of Genes and Genomes) pathways (Fig. 1E), suggesting enhanced phenolic biosynthesis was related to IB. Transcriptome analysis showed that the 11 differentially expressed genes (DEGs) for all six phenolic biosynthetic enzymes – phenylalanine ammonialyase (*AcPAL*), cinnamate 4-hydroxylase (*AcC4H*), 4-coumarate:CoA ligase (*Ac4CL*), hydroxycinnamoyl-CoA:shikimate/quinate hydroxycinnamoyl transferase (*AcHCT*), 5-O-(4-coumaroyl)-d-quinate 3′-monooxygenase (*AcC3′H*), and caffeoyl shikimate esterase (*AcCSE*) – were significantly upregulated in IB fruit vs healthy fruit (Fig. 1F). This is consistent with metabolomic analysis showing that the accumulation of six phenolic compounds was dramatically upregulated in pineapple with IB relative to non-stored fruit, particularly *p*-coumaric acid, *p*-coumaroyl shikimic acid, and caffeic acid (Fig. 1G). These results indicated that the phenolic biosynthesis pathway was activated in pineapple during storage, and the accumulation of phenolic compounds was highly correlated with IB development in pineapple.

### Screening for candidate transcription factors possibly involved in regulating phenolic biosynthesis

TFs are involved in various biological processes [10]. To unravel the transcription regulation mechanism of phenolic biosynthesis in pineapple during storage, transcriptomic analysis was used to identify differentially expressed transcription factors. As shown in Supplementary Data Fig. S1, five major TF families, i.e. *AcMYB*, *AcbHLH*, *AcWRKY*, *AcNAC*, and *AcbZip*, were identified in pineapples stored for 9 days compared with non-stored pineapples. Among them, the TF *AcbHLH144* showed the highest transcript abundance (Supplementary Data Fig. S1B–F). Previously, the bHLH TFs were found to be involved in the regulation of plant development processes [31], but it is not clear whether AcbHLH144 is involved in modulating plant tissue browning via regulating phenolic biosynthesis. Here we showed that *AcbHLH144* expression was negatively correlated with all the major genes involved in phenolic biosynthesis (Fig. 1F, Supplementary Data Fig. S1C), implying that AcbHLH144 may negatively regulate the biosynthesis of phenolic compounds in pineapple fruit during storage.

### Overexpression of *AcbHLH144* decreased phenolic accumulation in pineapple

**Figure 2:**
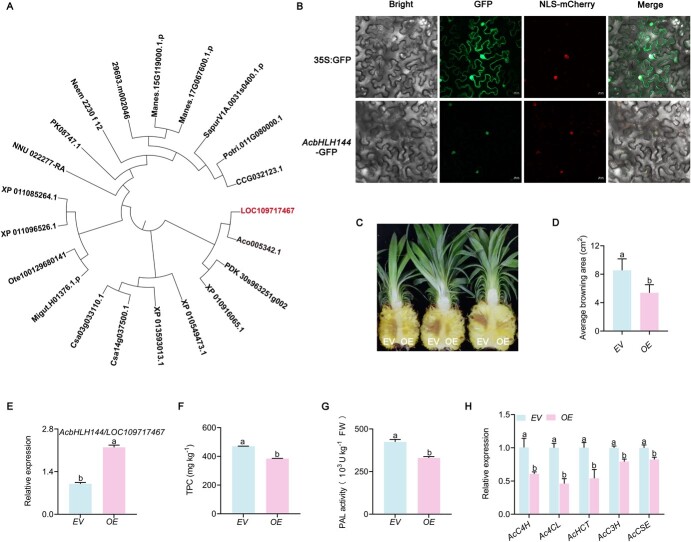
Overexpression of *AcbHLH144* TF in pineapple suppressed biosynthesis of phenolic compounds. **A** Phylogenetic relationship between AcbHLH144 and other plants (top 20). The bHLH marked in red is *bHLH144*. Aco005342.1, *Ananas comosus* (bHLH75); PDK_30s963251g002, *Phoenix dactylifera* (bHLH144); XP_010916065.1, *Elaeis guineensis* (bHLH144); XP_010549473.1, *Tarenaya hassleriana* (bHLH144-like); XP_013593013.1, *Brassica oleracea* (bHLH144); Csa14g037500.1, *Camelina sativa* (bHLH144-like); Csa03g033110.1, *Camelina sativa* (bHLH144); Migut.H01376.1.p, *Mimulus guttatus* (bHLH); Ote100129680141, *Ocimum tenuiflorum* (bHLH); XP_011096526.1, *Sesamum indicum* (bHLH144); XP_011085264.1, *Sesamum_indicum* (bHLH144); NNU_022277-RA, *Nelumbo nucifera* (bHLH144-like); PK08747.1, *Cannabis sativa* (bHLH144); Neem_2230_f_12, *Azadirachta indica* (bHLH); 29693.m002046, *Ricinus communis* (bHLH); Manes.15G119000.1.p, *Manihot esculenta* (bHLH144); Manes.17G067600.1.p, *Manihot esculenta* (bHLH144); SapurV1A.0031s0400.1.p, *Salix purpurea* (bHLH144); Potri.011G080000.1, *Populus trichocarpa* (bHLH144); CCG032123.1, *Populus euphratica* (bHLH144). The phylogenetic tree was produced by MEGAX with the neighbor-joining method and default parameters. The protein sequence was obtained from the Plant Transcription Factor Database (http://planttfdb.gao-lab.org/). **B** Subcellular localization of heterologously expressed AcbHLH144-GFP in leaves of *N. benthamiana*. mCherry staining was used to indicate the nucleus. Scale bar, 20 μm. **C** Phenotypes of pineapple resulting from transient expression of *AcbHLH144* in pineapple fruit. The photograph was taken 4 days after infiltration with *A. tumefaciens* harboring plasmid *pSuper:AcbHLH144* (right, OE) or empty vector (left, EV). **D** Size of the browning area was evaluated 4 days after infiltration. **E** Pineapple tissues surrounding the infiltration site were sampled for evaluation of the expression of *AcbHLH144*. **F** Total phenolic compounds (TPC). **G** PAL activity. **H** Expression of phenolic biosynthetic genes. Data are given as mean ± standard deviation (*n* = 3). Significant differences are shown by letters above the bars (*P* < .05).

To check whether AcbHLH144 TF functions in regulating phenolic biosynthesis, we cloned the coding sequence (CDS) of *AcbHLH144* from pineapple fruit. Phylogenetic analysis indicated that AcbHLH144 shared high similarity in amino acid sequence with Aco005342.1 from *A. comosus* (Fig. 2A, Supplementary Data Fig. S2). The transient overexpression of AcbHLH144-GFP fusion protein in *Nicotiana benthamiana* leaf tissues showed AcbHLH144 protein is localized to the nucleus (Fig. 2B), suggesting that AcbHLH144 functions in the nucleus, consistent with previous studies on the localization of bHLH TFs [13, 31, 32]. Then we investigated how AcbHLH144 regulated phenolic compound accumulation in pineapple fruit during storage. To this end, the *AcbHLH144* overexpression vector was transformed into pineapple fruit via *Agrobacterium tumefaciens* mediation. As shown in Fig. 2C, pineapple fruit with *pSuper 1300* (empty vector or EV) or *pSuper:AcbHLH144* (overexpression, OE) showed brown spots around the infection site, but the OE fruit had significantly smaller average browning area than the EV fruit (Fig. 2D)*.* Meanwhile, the OE pineapple displayed a significantly higher *AcbHLH144* transcription level than the EV fruit (Fig. 2E), suggesting the effective overexpression of *AcbHLH144* in pineapple. In addition, compared with EV fruit, OE fruit displayed reduced TPC contents (Fig. 2F), lower PAL activities (Fig. 2G), and decreased expression of five key genes in the phenolic biosynthetic pathway: *AcC4H*, *Ac4CL5*, *AcHCT4*, *AcC3′H*, and *AcCSE* (Fig. 2H). These results together strongly suggest that AcbHLH144 negatively regulates phenolic biosynthesis in pineapple fruit.

### Overexpression of *AcbHLH144* decreased phenolic accumulation in *Arabidopsis*

**Figure 3:**
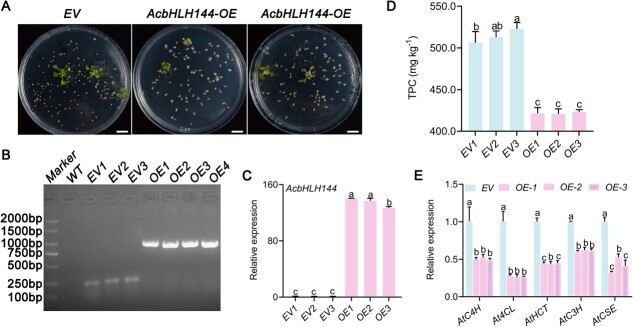
Overexpression of *AcbHLH144* decreased phenolic biosynthesis in *Arabidopsis* seedlings. **A** Screening for *T*_1_ seedlings overexpressing *AcbHLH144* on resistance plates. Scale bar = 1 cm. **B** PCR detection of *T*_1_ seedlings overexpressing *AcbHLH144*. **C** Expression of *AcbHLH144* gene in transgenic *Arabidopsis* lines. **D** Total phenolic content (TPC) in transgenic *Arabidopsis* lines. **E** Expression of major phenolic biosynthetic genes in *AcbHLH144*-overexpressing *Arabidopsis* lines. Significant differences are shown by letters above the bars (*P* < .05).

*Arabidopsis* lines that overexpress *AcbHLH144* (OE) and control lines (EV, empty vector) (Fig. 3A–C) were generated to verify AcbHLH144’s function in regulating phenolic biosynthesis (Fig. 3D and E). The OE lines had lower TPC contents (Fig. 3D) and decreased expression of *AtC4H*, *At4CL*, *AtHCT*, *AtC3H*, and *AtCSE* relative to the EV lines (Fig. 3E), confirming that AcbHLH144 negatively regulated phenolic biosynthesis in pineapple fruit.

### AcbHLH144 directly regulates expression of *Ac4CL5*

**Figure 4:**
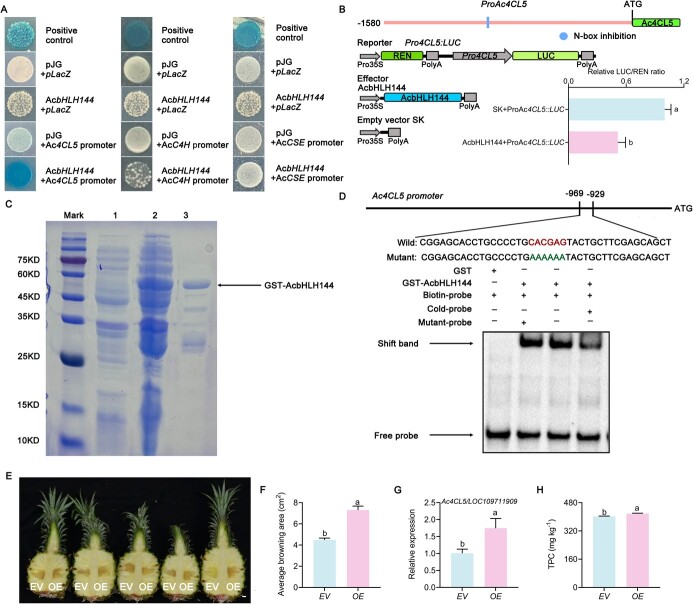
AcbHLH144 TF inhibited transcription of *Ac4CL5* (*LOC109711909*). **A** Y1H assay demonstrating binding of AcbHLH144 to the promoter of *Ac4CL5*. The *RhHB1* + *RhGA20ox1* promoter was used as a positive control. AcbHLH144 + pLacZ, pJG + pLacZ, pJG + *Ac4CL5* promoter, pJG + *AcCC4H* promoter and pJG + *AcCSE* promoter were used as negative controls. **B** Dual-luciferase reporter assay showing specific binding of AcbHLH144 to the promoter of *Ac4CL5*. (Top) Illustration of *cis* elements upstream of the *Ac4CL5* gene. (Bottom) Schematic illustration of the constructs for effector and reporter (left) and dual-luciferase activity (right). **C** GST-AcbHLH144 recombinant protein purification. Mark, marker; 1 and 2, *E. coli* strain with GST-AcbHLH144 plasmid before and after induction; 3, Purification of GST-AcbHLH144 recombinant protein. **D** EMSA demonstrating that AcbHLH144 specifically bound to the *Ac4CL5* promoter*.***E** Phenotypes of pineapple resulting from transient expression of *Ac4CL5* in pineapple fruit. The photograph was taken 4 days after infiltration with *A. tumefaciens* harboring plasmid *pSuper:Ac4CL5* (right, OE) or empty vector (left, EV). Scale bar = 1 cm. **F** Overexpression of *Ac4CL5* in pineapple fruit enhanced tissue browning. **G** Pineapple tissues surrounding the site of infiltration site were sampled for evaluation of expression of *Ac4CL5*. **H** TPC contents of pineapple overexpressing *Ac4CL5*. Significant differences are indicated by letters above the bars (*P* < .05).

To further characterize the role of AcbHLH144 in regulating biosynthesis of phenolics, the mode of action of AcbHLH144 TF was investigated. *AcC4H*, *Ac4CL5*, and *AcCSE* may be the target genes of AcbHLH144, as they showed expression patterns opposite to that of *AcbHLH144* (Fig. 2H, Supplementary Data Fig. S3). The yeast one-hybrid (Y1H) assay showed that AcbHLH144 directly bound to the promoter of *Ac4CL5*, but not to those of *AcC4H* and *AcCSE* (Fig. 4A). A dual-luciferase reporter (DLR) assay showed that AcbHLH144 specifically bound to the *Ac4CL5* promoter and suppressed its transcription (Fig. 4B). Notably, based on the different binding modes, bHLHs can be divided into three categories: some bind to the E-box/G-box (CANNTG), others bind to the N-box and the rest cannot bind to DNA [10]. As the N-box was found in the promoters of *Ac4CL5* and *AcCSE* (Fig. 4B, Supplementary Data Figs S4 and S6), but not in that of *AcC4H* (Supplementary Data Fig. S5), while the E-box/G-box was found in all three (Supplementary Data Figs S4–S6), we checked whether AcbHLH144 bound to the N-box of the *Ac4CL5* promoter to regulate its expression. The EMSA confirmed the specific binding of AcbHLH144 to the N-box (CACGAG) motifs in the *Ac4CL5* promoter (Fig. 4C and D). We then checked whether *Ac4CL5* was involved in phenolic biosynthesis by transient overexpression of *Ac4CL5* in pineapple fruit. As shown in Fig. 4E–G, the OE fruit had a significantly bigger browning area than the EV fruit*,* which is consistent with the enhanced *Ac4CL5* expression and higher TPC contents (Fig. 4H), suggesting *Ac4CL5* plays an important role in the biosynthesis of phenolic compounds. The results strongly indicated that AcbHLH144 directly bound to the *Ac4CL5* promoter, inactivated its transcription, and thereby suppressed phenolic biosynthesis.

### Exogenous abscisic acid reduced phenolic compound accumulation in pineapple fruit

**Figure 5:**
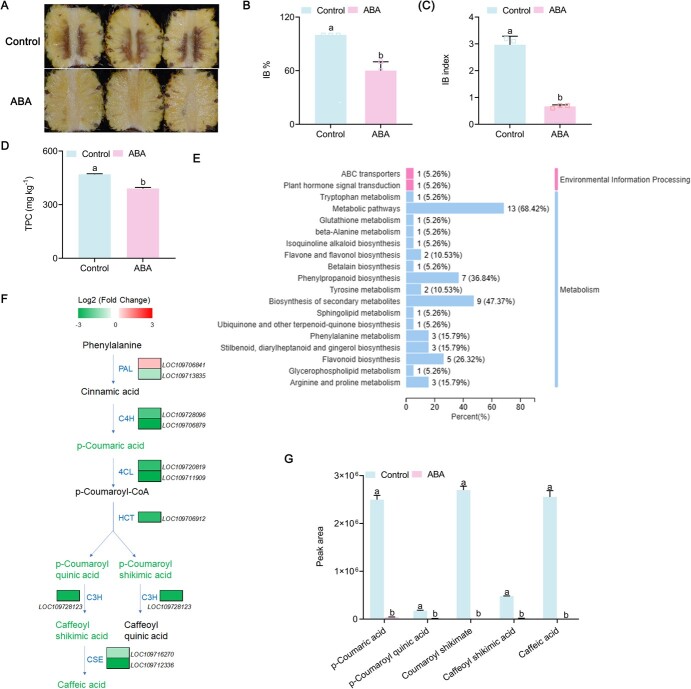
Changes in phenolic compounds in pineapples in response to ABA. **A** Pictures taken 9 days after treatment. Control, distilled water; ABA, 0.2 g L^−1^ ABA. **B** IB incidences. **C** IB index. **D** Total phenolic compounds (TPC). **E** The most enriched 19 KEGG pathways of differentially accumulated metabolites (DAMs) in ABA-treated fruit relative to the control. **F** Reconstructed phenolic biosynthetic pathway with DAMs and DEGs. Color in DEG charts: red, upregulation; green, downregulation. Font color for DAMs: green, downregulation; black, no significant change. **G** Relative abundance (peak area) of each of the five phenolic acids in pineapples. Significant differences are indicated by letters above the bars (*P* < .05).

As exogenous ABA alleviated IB in pineapple [1], we investigated whether and how ABA regulated *AcbHLH144*. We confirmed that ABA reduced IB severity and decreased TPC of pineapple fruit (Fig. 5A–D), and then showed that in ABA-treated pineapple the phenylpropanoid biosynthesis pathway was among the top three most enriched KEGG pathways in comparison with the control (Fig. 5E). Transcriptomic profiling revealed that nine DEGs for the six enzymes involved in phenolic compound biosynthesis, i.e. *AcPAL*, *AcC4H*, *Ac4CL*, *AcHCT*, *AcC3′H*, and *AcCSE*, were significantly downregulated in ABA-treated fruit (Fig. 5F). Metabolomic profiling revealed that the accumulation of five phenolic acids was significantly downregulated by ABA treatment, particularly *p*-coumaric acid, *p*-coumaroyl shikimic acid, and caffeic acid (Fig. 5G). These results indicated that ABA suppressed phenolic biosynthesis in pineapples.

### Exogenous abscisic acid inhibited phenolic biosynthesis in pineapple fruit via activating *AcbHLH144* transcription

**Figure 6:**
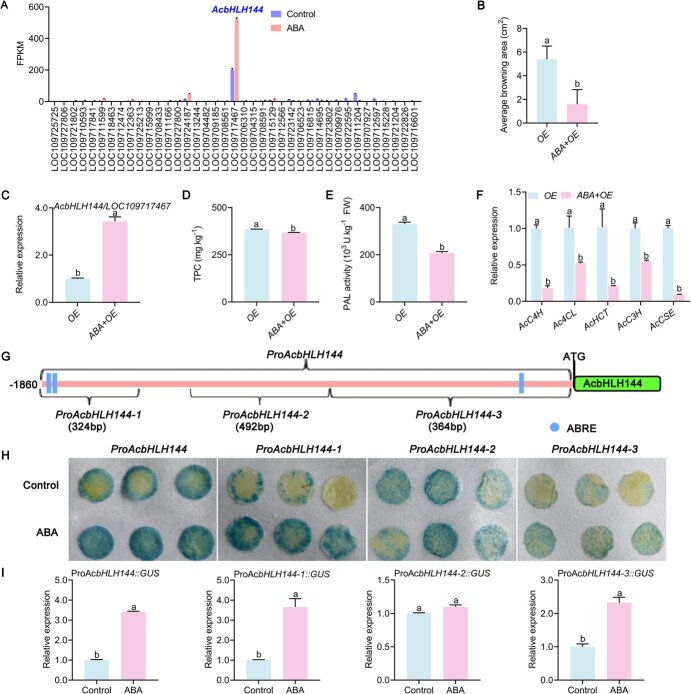
ABA suppressed phenolic biosynthesis in pineapple fruit via activating *AcbHLH144* transcription. **A** Expression (in terms of FPKM) of *AcbmHLH* TFs as influenced by ABA treatment. **B** Size of browning area was evaluated 4 days after infiltration of *pSuper:AcbHLH144*. **C** Expression of *AcbHLH144***. D** Total phenolic content (TPC). **E** PAL activity. **F** Expression of genes in the phenolic biosynthetic pathway was evaluated by quantitative RT–PCR. **G** Schematic illustration of the ABA-responsive element (ABRE) motifs in *cis* elements upstream of *AcbHLH144*. **H** GUS staining of leaves of tobacco transiently expressing the *GUS* gene driven by Pro*AcbHLH144*, Pro*AcbHLH144-1*, Pro*AcbHLH144-2*, and Pro*AcbHLH144-3*. **I** Expression of *GUS* in leaves of tobacco evaluated by RT–qPCR. Significant differences are shown by letters above the bars (*P* < .05).

As AcbHLH144 was shown to negatively modulate IB by suppressing *Ac4CL5* transcription (Fig. 4), we investigated how ABA regulated *AcbHLH144.* RNA-seq analysis showed that, among all the bHLH TFs, *AcbHLH144* was the highest in transcript abundance and was the most significantly upregulated by ABA (Fig. 6A). The upregulation of *AcbHLH144* by ABA was validated by RT–qPCR (Supplementary Data Fig. S7). To further elucidate the role of *AcbHLH144* in regulating phenolic accumulation in response ABA, an *AcbHLH144* overexpression vector was transformed into pineapple fruit following ABA treatment. The result showed that ABA reduced the browning area of the OE pineapples (Fig. 6B)*.* Moreover*,* compared with the OE, the ABA + OE displayed higher *AcbHLH144* expression, indicating the effectiveness of *AcbHLH144* overexpression and that ABA enhanced expression of *AcbHLH144* (Fig. 6C). TPC and PAL activity in the ABA + OE fruit were reduced relative to OE (Fig. 6D and E), which was consistent with the decreased expression of five phenolic biosynthetic genes, i.e. *AcC4H*, *Ac4CL5*, *AcHCT4*, *AcC3′H*, and *AcCSE*, in the ABA + OE fruit (Fig. 6F). These results strongly suggest that transcription of *AcbHLH144* was activated by ABA.

Sequence analysis of the promoter of *AcbHLH144* revealed three potential ABRE (ABA-responsive element) motifs [28] (Fig. 6G). To investigate whether transcription of *AcbHLH144* could be regulated by ABA, the *AcbHLH144* promoter sequence was divided into three fragments. The first fragment contained two potential ABRE motifs (Pro*AcbHLH144-1*) and the third contained one (Pro*AcbHLH144*-3), while the second contain none (Pro*AcbHLH144*-2). The whole sequence and different fragments of the *AcbHLH144* promoter were fused to the *GUS* reporter gene, respectively, before being transiently overexpressed in leaves of tobacco. As shown in Fig. 6H and I, ABA-treated tobacco leaves had higher enzyme activity and *GUS* expression driven by the whole sequence and fragments 1 and 3 of *AcbHLH144* promoter than did the control, indicating that ABA activated the transcription of *AcbHLH144*, while the activity and expression of *GUS* driven by fragment 2 showed no difference from the control. Taken together, the results revealed that ABA induced the transcription of *AcbHLH144* by recognizing the ABRE motif and thus inhibited phenolic biosynthesis, which ultimately led to control of IB.

## Discussion

Phenolic compound contents were previously shown to be positively correlated with browning of fruit and vegetables [2, 4, 8]. We got a similar result in this study with pineapple fruit that developed the IB symptom after 9 days of storage (Fig. 1A–D), confirming the link between phenolic compound accumulation and tissue browning. We further showed significant enrichment of the phenylpropanoid biosynthesis pathway in pineapples with IB (Fig. 1E), with all the six phenolic compounds in the phenolic biosynthesis pathway being dramatically enriched following 9 days of storage (Fig. 1F). This is consistent with the upregulation of 11 genes for the six enzymes involved in phenolic biosynthesis, i.e. *AcPAL*, *AcC4H*, *Ac4CL*, *AcHCT*, *AcC3′H*, and *AcCSE*, in fruit with IB (Fig. 1F), suggesting that the phenylpropanoid biosynthesis pathway was activated in harvested pineapples and that its activation may lead to IB. Interestingly, among the six differentially accumulated phenolic acids in fruit with IB, *p*-coumaric acid, *p*-coumaroyl shikimic, acid and caffeic acid displayed far greater increases than the other three (Fig. 1G), while the same three phenolic acids showed a similar extent of differences between ABA treatment and the control, except that they were downregulated by ABA (Fig. 5G), suggesting the three phenolic compounds play more important roles than others in regulating tissue browning. As *p*-coumaric acid is upstream of the other two, whether it is the most critical phenolic compound in the process of browning development remains to be investigated.

Previous research revealed that SmbHLH51 [33] positively regulates biosynthesis of phenolic compounds, while *SmbHLH60* [23] and *SmbHLH3* [27] function as negative regulators. In this study the role of AcbHLH144 in pineapple was characterized, as *AcbHLH144* displayed the highest transcript abundance among all the 180 TFs identified and was downregulated in fruit with IB (Supplementary Data Fig. S1). Transient overexpression of *AcbHLH144* in pineapples resulted in dramatically decreased phenolic biosynthesis (Fig. 2), which was in line with the results from ectopic overexpression of *AcbHLH144* in *Arabidopsis* (Fig. 3), suggesting AcbHLH144 as a repressor for phenolic synthesis in pineapple fruit during storage. Previous studies mostly addressed the role of bHLH TFs in regulating the biosynthesis of phenolic compounds as major metabolites in growing organs [13–15, 33–35]; this study for the first time showed that a bHLH TF can regulate tissue browning-related phenolic biosynthesis in fruit during postharvest processes.

bHLH TFs have been shown to bind to G-box/E-box sites of target genes to modulate plant responses to stresses. For example, CtbHLH41 directly binds to the promoter of *CtANS1* (G-box) to delay leaf senescence [36]. AtICE1 specifically binds to the promoter of *CBF3* (E-box) to induce *CBF3* transcription and improve cold resistance in *A. thaliana* [37]*.* In this study, the E-box/G-box site is predicted in promoters of the three phenolic biosynthetic genes, i.e. *AcC4H*, *Ac4CL5*, and *AcCSE* (Supplementary Data Figs S4–S6) that showed expression patterns opposite to that of *AcbHLH144* (Fig. 2H, Supplementary Data Fig. S3), but the Y1H assay showed that AcbHLH144 only bound to the *Ac4CL5* promoter, but not to *AcC4H* and *AcCSE* (Fig. 4A), suggesting the E-box/G-box may not be the binding site of *AcbHLH144* in the processes of regulating phenolic biosynthesis. The N-box (CACGAG) site was predicted in the promoter of *Ac4CL5* and *AcCSE* (Fig. 4B, Supplementary Data Figs S4 and S6), but not in *AcC4H* (Supplementary Data Fig. S5). Moreover, the EMSA confirmed that AcbHLH144 specifically bound to the N-box motif (Fig. 4C and D) and the DLR assay demonstrated a lower LUC/REN ratio in the presence of AcbHLH144 relative to the empty control. These results together suggest that AcbHLH144 suppressed *Ac4CL5* via binding to the N-box (Fig. 4B). Suppression of *Pb4CL2* expression resulted in lignin decreases [38]. Overexpression and antisense of *Sm4CL2* promotes or suppresses salvianolic acid biosynthesis in *S. miltiorrhiza* hairy roots [39]. In this study, transient overexpression of *Ac4CL5* in pineapples resulted in increased phenolic biosynthesis and a bigger browning area (Fig. 4E–H), suggesting an important role of *Ac4CL5* in the biosynthesis of phenolic compounds in pineapple. These results together indicated that AcbHLH144 suppressed phenolic biosynthesis via inactivating *Ac4CL5* transcription in pineapple fruit during storage. It is reasonable to speculate that *AcC4H* and *AcCSE*, which function upstream and downstream of and both showed a similar trend to *Ac4CL5*, may also play substantial roles in phenolic biosynthesis, but how they are transcriptionally regulated to coordinate with *Ac4CL5* in modulating phenolic biosynthesis in pineapple during storage remains to be elucidated.

To further verify the role of AcbHLH144 in regulating *Ac4CL5*, we provided a context opposite to that of pineapple developing IB symptoms by exogenous application of ABA, as the latter can reduce TPC and alleviate IB severity [1], which was confirmed in this study (Fig. 5A–D). Through a metabolomic approach we showed that the phenylpropanoid biosynthesis pathway was among the top three most enriched KEGG pathways in pineapples exposed to ABA (Fig. 5E). In addition, the major phenolic acids were reduced and key genes in the phenylpropanoid biosynthesis pathway were downregulated in response to ABA (Fig. 5F and G). These results suggest that the phenolic biosynthesis pathway was inactivated by ABA treatment. Among the total of 39 bHLH TFs identified in ABA-treated fruit vs the control, *AcbHLH144* transcription was the highest and was dramatically enhanced by ABA (Fig. 6A), suggesting that *AcbHLH144* can be induced by ABA. Transient overexpression of *AcbHLH144* in pineapple fruit exposed to ABA treatment reduced TPC and PAL activity and downregulated key phenolic biosynthetic genes (Fig. 6B–F), further confirming that AcbHLH144 as the repressor for phenolic biosynthesis in pineapple was induced by ABA. As sequence analysis of the promoter of *AcbHLH144* revealed three potential ABRE motifs (Fig. 6G), experiments were conducted to verify whether ABA could enhance transcription of *AcbHLH144*. When the whole promoter sequence of *AcbHLH144* and each of the three fragments of the promoter were used to drive the *GUS* reporter gene, respectively, ABA enhanced expression of *GUS* driven by the whole promoter sequence of *AcbHLH144* and by the two promoter fragments that contained ABRE motifs, but it did not induce expression of *GUS* driven by the *AcbHLH144* promoter fragment without ABRE motifs (Fig. 6H and I). These results, together with the transient overexpression of *AcbHLH144* in pineapple and the ectopic overexpression of *AcbHLH144* in *Arabidopsis*, confirmed that AcbHLH144 negatively regulated phenolic biosynthesis in pineapple by inactivating *Ac4CL5* transcription, and indicated that ABA activated *AcbHLH144* expression. Previous studies showed that *AtbHLH129* gene expression is upregulated by exogenous ABA to promote root elongation [40] and the bHLH TF SlPRE2 regulates tomato development and modulates plant response to gibberellin [41]. SmbHLH60 regulates phenolic acids in *S. miltiorrhiza* via binding to *SmTAT1* in response to the MeJA signal [23]. To the authors’ knowledge, this is the first work showing that ABA can suppress phenolic biosynthesis and inhibit tissue browning via activating a bHLH TF.

In conclusion, this work showed AcbHLH144 TF as a repressor for phenolic biosynthesis in pineapple fruit. AcbHLH144 negatively regulated *Ac4CL5* transcription to suppress phenolic compound accumulation and IB occurrence. ABA activated AcbHLH144 transcription to suppress phenolic biosynthesis and thereby reduce IB. The findings provide novel insight into the mechanism of the AcbHLH144 TF in regulating pineapple IB in postharvest processes.

## Materials and methods

### Plant material

Pineapple (*Ananas comosus* L. Merr.) fruits were harvested at 70% commercial maturity stage from a commercial plantation in Xuewen County, Zhanjiang City, and were transported to the Postharvest Pathology and Molecular Biology Laboratory in the College of Horticulture, South China Agricultural University. Fruits uniform in size and disease-free were used as experimental material. All fruits were washed and air-dried before being sprayed with ABA solution (0.2 g L^−1^) or distilled water (control) until runoff and air-dried. Samples of pulps were collected every 3 days, frozen in liquid nitrogen, and stored at −80°C.

The ABA-treated and control pineapples were used for a transient expression test. One side of the pineapple was injected at the equatorial line with empty vector *pSuper1300* (EV) and the other side was injected with *pSuper1300:AcbHLH144* (OE). There were three replications for each treatment, which contained 15 fruits. Fruits were wrapped in perforated polyethylene film with a thickness of 0.03 mm and placed in a dark environment of 20 ± 1°C and 95% RH. Samples of pulps were harvested at 4 days for analysis.

Tobacco (*Nicotiana benthamiana*) and *Arabidopsis* plants were grown under 16 h of light and 8 h of darkness at the temperature of 22°C.

### Assessment of internal browning incidence and index

IB incidence and index were assessed according to methods described previously [1].

### Total phenolic content assay

The TPC was evaluated as previously described with minor modification [1]. The sample (0.5 g) was incubated in 60% ethanol solution (2.5 mL) at 60°C for 2 h and centrifuged at 13 000 rpm for 20 min. Then, the solution containing supernatant (0.1 mL), double-distilled water (0.9 mL), Folin–phenol reagent (1 mL, 0.2 M), and sodium carbonate solution (0.7 mL, 1 M) was added to a new test tube, which was incubated for 30 min (25°C). Then, absorbance (765 nm) was determined and TPC was expressed as milligrams of gallic acid equivalent per gram of fresh sample.

### Metabolomic analysis

Samples from pineapple pulps were used to perform metabolomic analysis following the methods described previously [42]. VIP (variable importance in projection) ≥1 and absolute log_2_ fold change ≥1 was set as the threshold for significantly accumulated metabolites.

### Transcriptomic analysis

Pulp tissue samples of pineapple were used for RNA-seq analysis following the methods described previously [42].

### Subcellular localization in *N. benthamiana*

The open reading frame (ORF) of *AcbHLH144* without the stop codon was inserted into the Super1300 vector with the sequence (at the 5′ end) encoding green fluorescent protein (GFP) to construct the pSuper:AcbHLH144*-GFP* plasmid. The *AcbHLH144* gene’s subcellular localization was determined as previously described [6, 43]. *Agrobacterium* cells (GV3101) expressing pSuper:AcbHLH144*-GFP* were resuspended in solution (10 mM MES-KOH, 10 mM MgCl_2_, 200 μM acetosyringone, pH 5.6) to OD_600_ = 0.8, and infiltrated into *N. benthamiana* leaves. Fluorescence signals were observed 48 h later using a confocal laser-scanning microscope (Nikon, Japan). Primers are shown in Supplementary Data Table S1.

### Vector construction for *AcbHLH144* transient expression in pineapple fruit

The *AcbHLH144* (*LOC109717467*) core CDS was cloned using cDNAs from pineapple fruit and sequenced by Sangon Biotech (Shanghai) Co., Ltd. A 723-bp fragment of the *AcbHLH144* CDS was cloned into pSuper 1300 vector. Transient expression of *AcbHLH144* in pineapple fruit was performed as described previously [38]. The primer sequences are shown in Supplementary Data Table S1.

### Assessment of tissue browning in pineapple fruit transiently overexpressing *AcbHLH144*

To evaluate the browning area of pineapple resulting from transient expression of *AcbHLH144*, the pineapple was cut lengthwise along the injection hole, the length and width of the browning parts were determined, and the average browning area of the cut surface was calculated.

### RT–qPCR analysis

Total RNA was isolated from pineapple pulps as previously described [6]. qPCR was executed using the iScript cDNA Synthesis Kit (Bio-Rad, USA). SYBR Green Supermix (Bio-Rad, USA) was used for RT–qPCR reactions. *AcActin* (GenBank HQ148720.1) was used as the housekeeping reference gene. The reactions were run in the CFX96™ Optics Module (Bio-Rad, USA). Three replicates of each sample were analyzed. Relative expression of genes was calculated with the 2^−ΔΔCt^ algorithm. Primer sequences are shown in Supplementary Data Table S1.

### Phenylalanine ammonialyase activity assay

PAL activity was determined according to methods described previously [1].

### Transformation of *Arabidopsis*

A CDS fragment of *AcbHLH144* was ligated into the vector (pSuper 1300) before the latter was introduced into the cells (GV3101). Positive transgenic *Arabidopsis* lines (overexpressing *AcbHLH144*) were generated following methods described previously [6]. RT–qPCR was carried out to determine the expression of genes *AcbHLH144*, *AtC4H*, *At4CL*, *AtHCT*, *AtC3H*, and *AtCSE* in the transgenic lines. Primer sequences are shown in Supplementary Data Table S1.

### Yeast one-hybrid assay

The ORF fragment of *AcbHLH144* was fused to pJG4-5 vector (Clontech Laboratories, Inc., Mountain View, CA, USA) using XhoI and EcoRI sites, resulting in the GAD-*AcbHLH144* construct. The promoters of *AcC4H*, *Ac4CL5*, and *AcCSE* were ligated into pLacZi2μ vector, respectively. The Y1H assay was performed following the method previously described [43]. The combination (*RhHB1* + *RhGA20ox1* promoter) was used as a positive control. Primer sequences for Y1H are shown in Supplementary Data Table S1.

### Dual-luciferase reporter assay

The *AcbHLH144* ORF fragment was fused to pGreen II 62-SK vector to obtain the effector and the *Ac4CL5* promoter fragment was fused to pGreen II 0800-LUC vector to construct the reporter. *Agrobacterium* GV3101 (pSoup) including the effector and reporter was evenly mixed in a volume ratio of 8:2, and the mixture was co-infiltrated into the leaves of tobacco. The combination (pGreen II 62-SK + ProAc4CL5:LUC) was applied to the control group. Firefly and *Renilla* luciferases were determined 72 h after injection using a Dual Luciferase Reporter Gene Assay Kit (Yeasen, Shanghai, China). Primers for the DLR assay are shown in Supplementary Data Table S1.

### Electrophoretic mobility shift assay

The *AcbHLH144* ORF was fused to pGEX-4 T-1 vector to establish the GST- AcbHLH144 plasmid, before being transferred to cells of *Escherichia coli* strain Rosetta (DE3). Isopropyl-β-d-thiogalactopyranoside (0.5 M) was used as an inducer, and the cells were incubated at 16°C for 12 h. Then, the fusion protein (GST- AcbHLH144) was purified with a GST 4FF Sefinose™ Resin Kit (Sangon Biotech, Shanghai, China). The probe harboring the putative bHLH binding site N-box (CACGAG) originating from the Ac4CL5 promoter sequence was labeled with biotin using an EMSA Probe Biotin Labeling Kit (Beyotime, Shanghai, China). The DNA-binding assay was conducted with a Chemiluminescent EMSA Kit (Beyotime). The primers for EMSA are shown in Supplementary Data Table S1.

### Detection of Pro*AcbHLH144* responsiveness to abscisic acid

The *AcbHLH144* promoter was fused to vector (pCAMBIA1391), including the reporter gene of β-glucuronidase (GUS). The GUS transient test was performed as previously described [6]. Distilled water (control) and ABA (0.2 g L^−1^) solution were evenly sprayed on the leaves of tobacco 6 h after injection, respectively. Two days after treatment, the leaves of tobacco were stained according to the instructions in the GUS Staining Kit (Ready-To-Use). Total RNA of tobacco leaves was extracted and GUS reporter gene expression was detected by RT–qPCR as described in section RT-qPCR analysis. Primers are shown in Supplementary Data Table S1.

### Statistics

Data were analyzed with GraphPad Prism software version 8.0 (GraphPad Software, Inc., La Jolla, CA, USA). The mean ± standard deviation was used to represent the results. Data were analyzed using one-way analysis of variance (ANOVA). DPS software (version 9.01) was used for statistical analysis (*P* < .05) of Duncan's multiple range test.

## Supplementary Material

Web_Material_uhad185

## Data Availability

All relevant data in this study are provided in the article and its supplementary files. Accession numbers of genes found in this article: *AcbHLH144* (LOC10971747), *AcC4H* (LOC109706879), *Ac4CL* (LOC109711909), *AcHCT* (LOC109706912), *AcC3H* (LOC109728123), *AcCSE* (LOC1097112336), *AcActin* (GenBank: HQ148720.1) *AcbHLH75* (Aco005342.1), *PdbHLH144* (PDK_30s963251g002), *EgbHLH144* (XP_010916065.1), *ThbHLH144-like* (XP_010549473.1), *BobHLH144* (XP-013593013.1), *CsbHLH144-like* (Csa14g037500.1), *CsbHLH144* (Csa03g033110.1), *MgbHLH* (Migut.H01376.1.p), *OtbHLH* (Ote100129680141), *SibHLH144* (XP_011096526.1), *SibHLH144* (XP_011085264.1), *NnbHLH144-like* (NNU_022277-RA), *CsbHLH144* (PK08747.1), *AibHLH* (Neem_2230_f_12), *RcbHLH*-(29693.m002046), *MebHLH144* (Manes.15G119000.1.p), *Me*bHLH144 (Manes.17G067600.1.p), *SpbHLH144* (SapurV1A.0031s0400.1.p), *PtbHLH144* (Potri.011G080000.1), *PebHLH144* (CCG032123.1).
