# Supplementary material for: AcbHLH144 transcription factor negatively regulates phenolic biosynthesis to modulate pineapple internal browning
Source: Hortic Res. 2023 Sep 7;10(10):uhad185. doi: 10.1093/hr/uhad185 (PMC10611554; doi:10.1093/hr/uhad185)
Supplement: Web_Material_uhad185 [file web_material_uhad185.zip › Supplementary materials R1.docx]

**Supplementary materials**

**Table S1.** Sequences of primers used in this study.

| Assay | Gene name | Primer name | Primer sequence |
| --- | --- | --- | --- |
| qRT-PCR | *AcActin* | *AcActin-F* | CTGAGGAGCACCCTGTCTTG |
|  |  | *AcActin-R* | GCATAGAGGGAGAGCACAGC |
|  | *AcbHLH144* | *AcbHLH144-F* | CAGCCTTTGGAAAACTGCCC |
|  |  | *AcbHLH144-R* | AATTGGGCGAACCGAGCTTA |
|  | *AcC4H* | *AcC4H -F* | GACCTGCAATCCAGAAGGCT |
|  |  | *AcC4H -R* | CGTTCTTCTCCGCTTCGAGT |
|  | *Ac4CL5* | *Ac4CL5-F* | GCTCGTCGAGAGGTACAAGG |
|  |  | *Ac4CL5-R* | TATCACGAGCCGAACCGATG |
|  | *AcHCT4* | *AcHCT4-F* | TACACGAGCGCGATCTCAAA |
|  |  | *AcHCT4-R* | CTACCGCATACGTGGCTGAT |
|  | *AcC3H* | *AcC3H-F* | AAGAACACCTCTCCAGCGTG |
|  |  | *AcC3H-R* | TCCTTGCTCGTCAATCACCC |
|  | *AcCSE* | *AcCSE-F* | CTTCACCCAGTCCTTCCACC |
|  |  | *AcCSE-R* | GTAGGCCATGGCGATCTTCT |
|  | *AtC4H* | *AtC4H-F* | GGAAACGTTTGCAATTGATGATGT |
|  |  | *AtC4H-R* | GCAATTCCCCACTCGATAGACCAC |
|  | *At4CL* | *At4CL-F* | CTAATGCCAAACTCGGTCAGGGATAC |
|  |  | *At4CL-R* | CTCTTGTAAAACACAACCTGTTTCGAC |
|  | *AtHCT* | *AtHCT-F* | CTTCTCGTTTTGCAGGTGACTTTC |
|  |  | *AtHCT-R* | GTATTCTCAGGTCCTGATTTAG |
|  | *AtC3H* | *AtC3H-F* | CATTGGTCTTCTATGGGATATG |
|  |  | *AtC3H-R* | CTTCGGTGAGGTAGCATTAGA |
|  | *AtCSE* | *AtCSE-F* | CCGATGGTATCCGCTGCTACATGGGTG |
| Assay | Gene name | Primer name | Primer sequence |
|  |  | *AtCSE-R* | TTGTTCCCACTCTAGGCTTCCCTGTATATCTTTG |
|  | *AtActin* | *AtActin-F* | CCTCAGCACATTCCAGCAGATGT |
|  |  | *AtActin-R* | TGGGACTAAAACGCAAAACGAAAG |
| Subcellular localization | *AcbHLH144-GFP* | *AcbHLH144-GFP-F* | CCAAATCGACTCTAGTCTAGAATGCAGGGGGACGCGAGA |
|  |  | *AcbHLH144-GFP-R* | CCCGGGCCCCTGCAGAAGCTTTATCGCAGCTTTGGTCATCAAA |
| Overexpression assay | *AcbHLH144-GFP* | *AcbHLH144-GFP-F* | CCAAATCGACTCTAGTCTAGAATGCAGGGGGACGCGAGA |
|  |  | *AcbHLH144-GFP-R* | CCCGGGCCCCTGCAGAAGCTTTATCGCAGCTTTGGTCATCAAA |
| Dua-luciferase reporter assay | *AcbHLH144-62-SK* | *AcbHLH144-62-SK-F* | AGAACTAGTGGATCCCCCGGGATGCAGGGGGACGCGAGAAT |
|  |  | *AcbHLH144-62-SK-R* | GATAAGCTTGATATCGAATTCATCGCAGCTTTGGTCATCAAAG |
|  | *0800-Ac4CL5pro* | *0800-Ac4CL5pro-F* | GTCGACGGTATCGATAAGCTTTTCTCAAAGCGCTGTGAGCT |
|  |  | *0800-Ac4CL5pro-R* | AGAACTAGTGGATCCCCCGGGCACCCCCTTCGGCAGC |
| Y1H | *AcbHLH144-pJG4-5* | *AcbHLH144-pJG4-5-F* | AGATTATGCCTCTCCCGAATTCATGCAGGGGGACGCGAGAAT |
|  |  | *AcbHLH144-pJG4-5-R* | AGAAGTCCAAAGCTTCTCGAGTCAATCGCAGCTTTGGTCATCAAAG |
|  |  | *pLacZi2µ-Ac4CL5pro-F* | CTTTGATATTGGATCGAATTCTTCTCAAAGCGCTGTGAGCT |
|  |  | *pLacZi2µ-Ac4CL5pro-R* | ATACAGAGCACATGCCTCGAGGGTCAGCATCACCCCCTTC |
|  |  | *pLacZi2µ-AcC4Hpro-F* | CTTTGATATTGGATCGAATTCCTCCTGCTTTCCATGCCCTT |
|  |  | *pLacZi2µ-AcC4Hpro-R* | ATACAGAGCACATGCCTCGAGGATAGCCATTTTCATCAATT |
|  |  | *pLacZi2µ-AcCSEpro-F* | CTTTGATATTGGATCGAATTCAGAGGAAGTGCCAGGAAGTG |
|  |  | *pLacZi2µ-AcCSEpro-R* | ATACAGAGCACATGCCTCGAGGGGATGAGCCATGAGGAGAA |
| EMSA | *GST-AcbHLH144* | *GST-AcbHLH144-F* | GATCTGGTTCCGCGTGGATCCATGCAGGGGGACGCGAGAAT |
|  |  | *GST-AcbHLH144-R* | GATCTGGTTCCGCGTGGATCCATGCAGGGGGACGCGAGAAT |
|  |  | *EMSA-Ac4CL5pro-F* | CGGAGCACCTGCCCCTGCACGAGTACTGCTTCGAGCAGCT |
|  |  | *EMSA- Ac4CL5pro-R* | AGCTGCTCGAAGCAGTACTCGTGCAGGGGCAGGTGCTCCG |
|  |  | *EMSA-mAc4CL5pro-F* | CGGAGCACCTGCCCCTGAAAAAATACTGCTTCGAGCAGCT |
|  |  | *EMSA-mAc4CL5pro-R* | AGCTGCTCGAAGCAGTAAAAAAACAGGGGCAGGTGCTCCG |
| GUS | *pCAMBIA1391-Ac bHLH144* | *pCAMBIA1391-Ac bHLH144-F* | TGGGCCCGGCGCGCCAAGCTTTTGCCACACGTGCGAAAT |
|  |  | *pCAMBIA1391-Ac bHLH144-R* | CGGGGATCCGTCGACCTGCAGCCCCTGCATGGGTCTATAGT |
| Assay | Gene name | Primer name | Primer sequence |
|  | *pCAMBIA1391-Ac bHLH144-1* | *pCAMBIA1391-Ac bHLH144-1-F* | TGGGCCCGGCGCGCCAAGCTTTTGCCACACGTGCGAAAT |
|  |  | *pCAMBIA1391-Ac bHLH144-1-R* | CGGGGATCCGTCGACCTGCAGGGGAAAAAGGGAGCGCTTTT |
|  | *pCAMBIA1391-Ac bHLH144-2* | *pCAMBIA1391-Ac bHLH144-2-F* | TGGGCCCGGCGCGCCAAGCTTTGGAGGAGGTCGGCGAAATA |
|  |  | *pCAMBIA1391-Ac bHLH144-2-R* | CGGGGATCCGTCGACCTGCAGACGAAAGCATGCAGGCCATC |
|  | *pCAMBIA1391-Ac bHLH144-3* | *pCAMBIA1391-Ac bHLH144-3-F* | TGGGCCCGGCGCGCCAAGCTTTGGCCTGCATGCTTTCGT |
|  |  | *pCAMBIA1391-Ac bHLH144-3-R* | CGGGGATCCGTCGACCTGCAGCCCCTGCATGGGTCTATAGT |


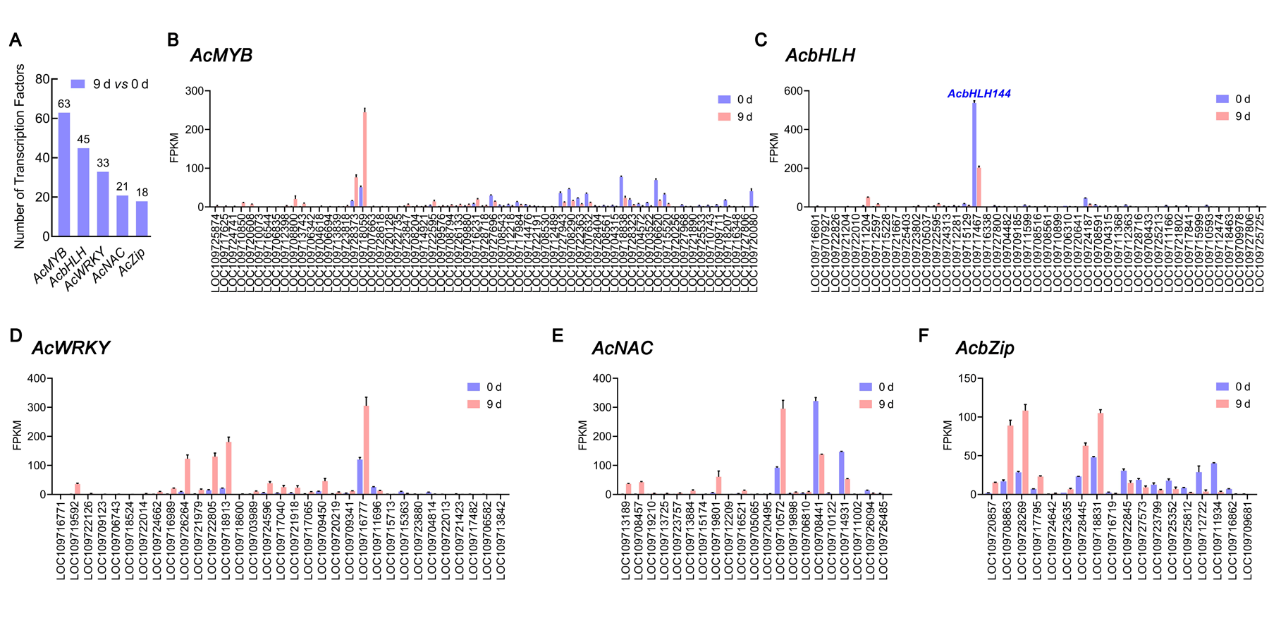


**Figure S1.** Change of differentially expressed transcription factors (DETFs) in pineapple fruit with IB symptoms. **A:** The number of different TF families, *AcMYB*, *AcbHLH*, *AcWRKY*, *AcNAC* and *AcbZip*, identified from pineapples stored for 9 days in comparison to fruit before storage. **B-F:** The patterns of expression (FPKM) of five different TF familiesm *AcMYB*, *AcbHL*, *AcWRKY*, *AcNA* and *AcbZi*.


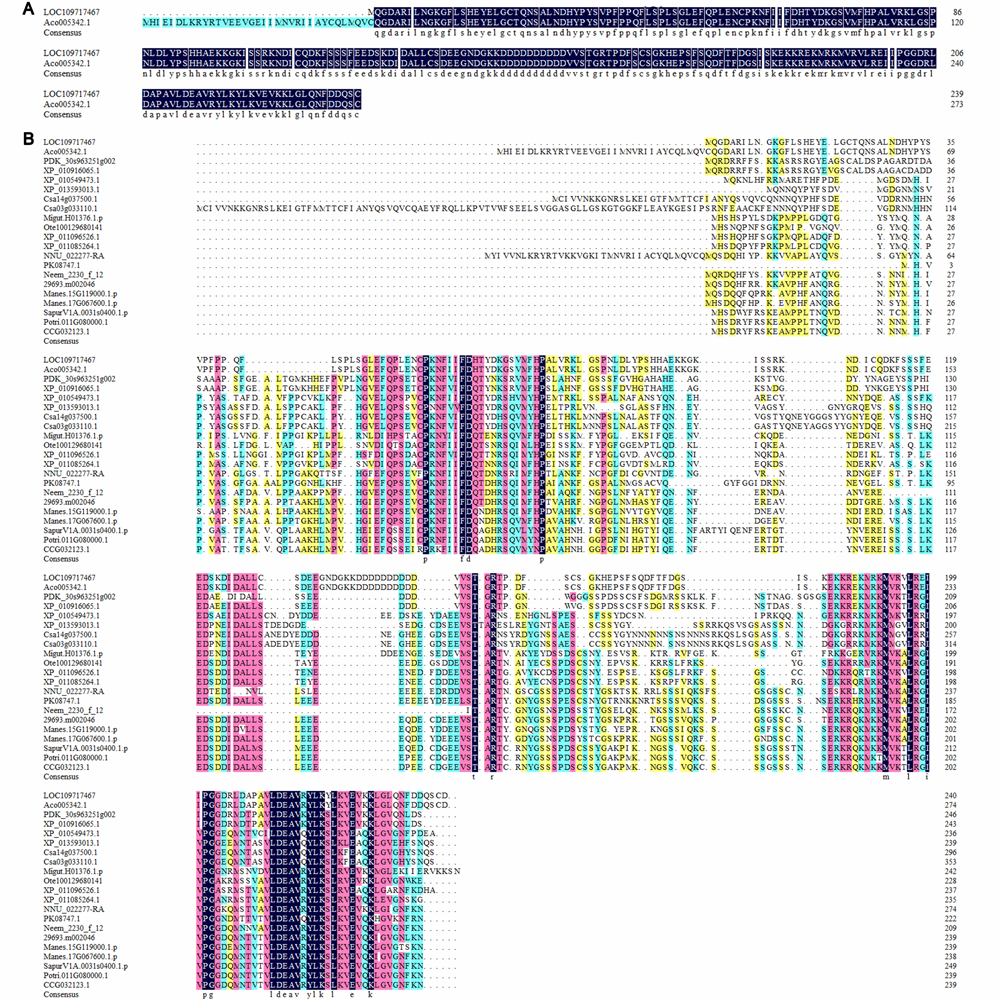


**Figure S2.** Amino acid alignment. **A:** Amino acid alignment of *LOC109717467* (AcbHLH144) with Aco005342.1. **B:** Amino acid alignment of *LOC109717467* (AcbHLH144) with other bHLHs (top 20): Aco005342.1, PDK_30s963251g002, XP_010916065.1, XP_010549473.1, XP_013593013.1, Csa14g037500.1, Csa03g033110.1, Migut.H01376.1.p, Ote100129680141, XP_011096526.1, XP_011085264.1, NNU_022277-RA, PK08747.1, Neem_2230_f_12, 29693.m002046, Manes.15G119000.1.p, Manes.17G067600.1.p, SapurV1A.0031s0400.1.p, Potri.011G080000.1 and CCG032123.1. The protein sequences were obtained from Plant Transcription Factor Database (<http://planttfdb.gao-lab.org/>).


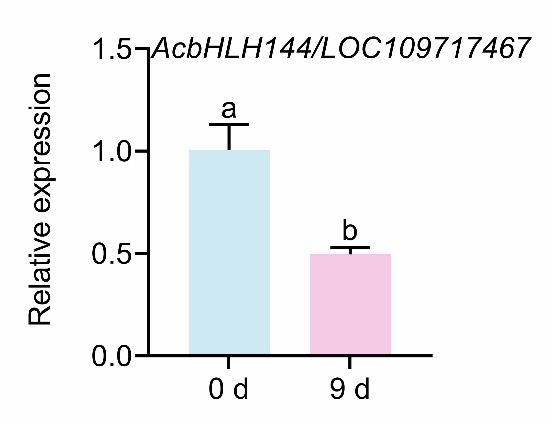


**Figure S3.** The expression of *AcbHLH144* in pineapple stored for 9 days in comparison to fruit before storage evaluated by RT-qPCR. Data are given as mean ± SD (n=3). Significance of differences is indicated by letters above the bars (*P*<0.05).


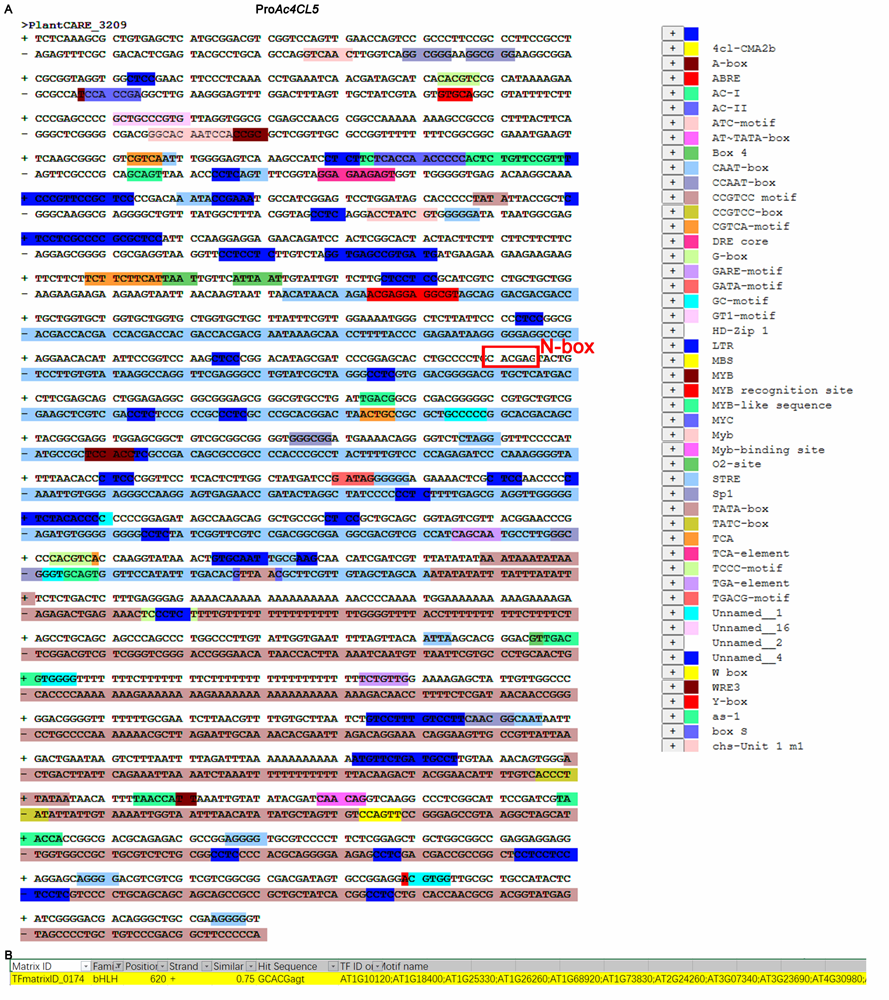


**Figure S4.** The promoter of *Ac4CL5*. **A:** The promoter of *Ac4CL5* was analyzed by PlantCARE ([PlantCARE, a database of plant promoters and their cis-acting regulatory elements (ugent.be)](http://bioinformatics.psb.ugent.be/webtools/plantcare/html/)) software. **B:** The promoter of *Ac4CL5* was analyzed by [PlantPAN 2.0 (ncku.edu.tw)](http://plantpan2.itps.ncku.edu.tw/) software.


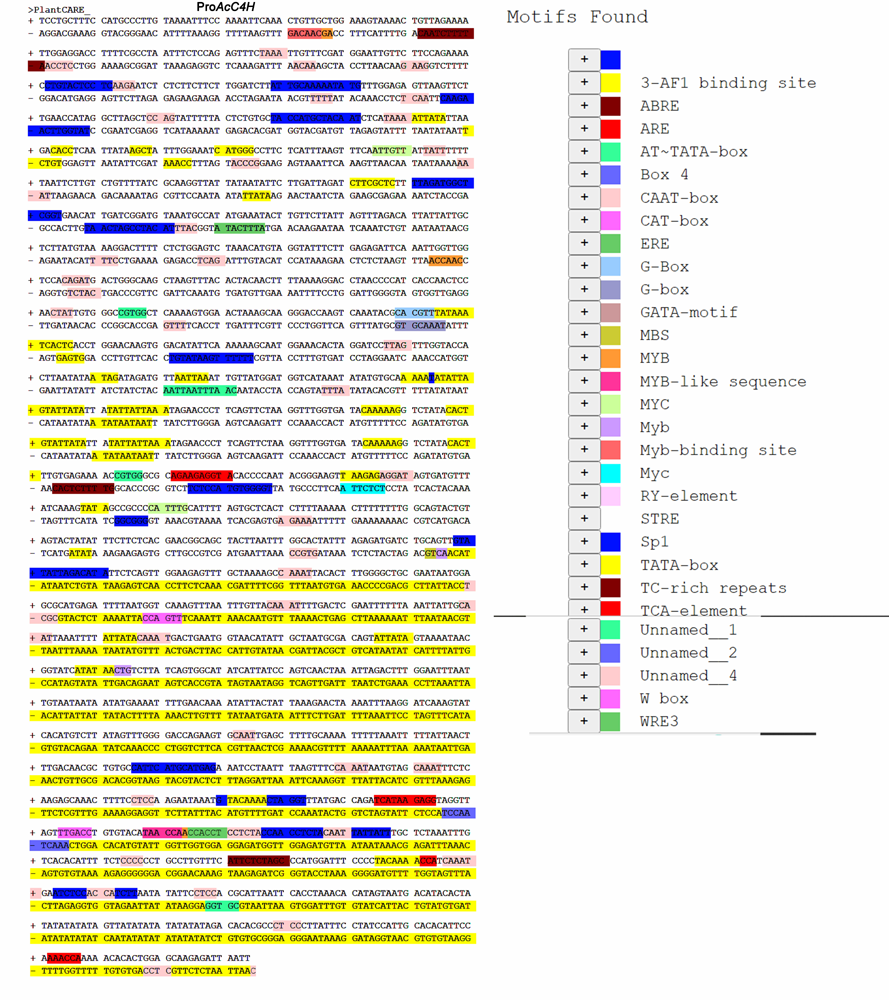


**Figure S5.** The promoter of *AcC4H* was analyzed by PlantCARE ([PlantCARE, a database of plant promoters and their cis-acting regulatory elements (ugent.be)](http://bioinformatics.psb.ugent.be/webtools/plantcare/html/)) software.


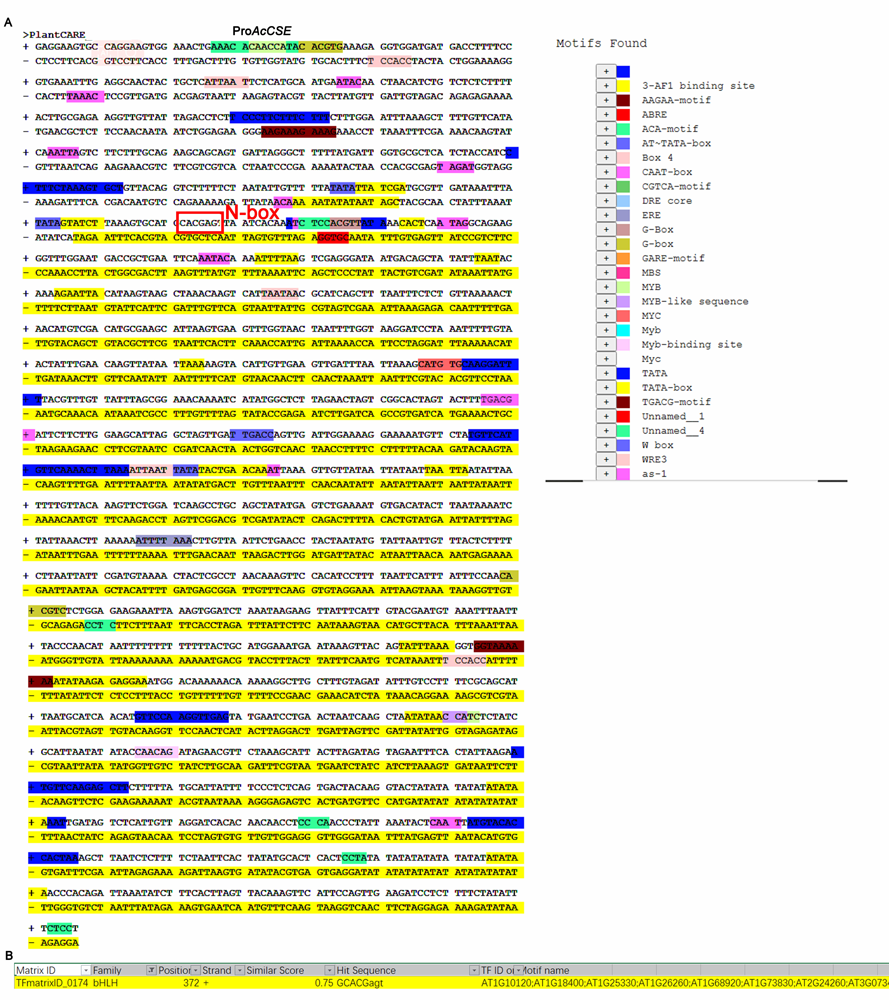


**Figure S6.** The promoter of *AcCSE*. **A:** The promoter of *AcCSE* was analyzed by PlantCARE ([PlantCARE, a database of plant promoters and their cis-acting regulatory elements (ugent.be)](http://bioinformatics.psb.ugent.be/webtools/plantcare/html/)) software. **B:** The promoter of *AcCSE* was analyzed by [PlantPAN 2.0 (ncku.edu.tw)](http://plantpan2.itps.ncku.edu.tw/) software.


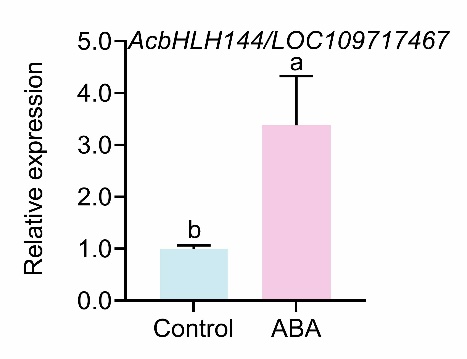


**Figure S7.** The expression of *AcbHLH144* in ABA-treated pineapple in comparison to the control evaluated by RT-qPCR. Data are given as mean ± SD (n=3). Significance of differences is indicated by letters above the bars (*P*<0.05).
